# Validation of Finnish Neecham Confusion Scale and Nursing Delirium Screening Scale using Confusion Assessment Method algorithm as a comparison scale

**DOI:** 10.1186/s12912-016-0199-6

**Published:** 2017-01-19

**Authors:** Satu Poikajärvi, Sanna Salanterä, Jouko Katajisto, Kristiina Junttila

**Affiliations:** 1 Department of Nursing Science, Faculty of Medicine, University of Turku, Turku, Finland; 2 Helsinki University Hospital, Perioperative, Intensive Care and Pain Medicine, PO Box 266, Helsinki, FI00029 HUS Finland; 3 Department of Nursing, Turku University Hospital, Turku, Finland; 4 Department of Mathematics and Statistics, Faculty of Mathematics and Natural Sciences, University of Turku, Turku, Finland; 5 Helsinki University Hospital, Group Administration, Helsinki, Finland

**Keywords:** Confusion, Confusion Assessment Method, Delirium, Instrument testing, Neecham Confusion Scale, Nursing Delirium Screening Scale

## Abstract

**Background:**

Delirium is a common clinical problem with acute and fluctuating onset. Early notification of its symptoms can lead to earlier detection and management of this state. Valid and reliable instruments are required for successful nursing practice. The purpose of the study was to psychometrically test the Finnish versions of the Neecham Confusion Scale (NEECHAM) and the Nursing Delirium Screening Scale (Nu-DESC) in surgical nursing care, utilizing the Confusion Assessment Method (CAM) algorithm as a comparison scale.

**Methods:**

This randomized, blinded, instrument testing study was conducted at one university hospital in one surgical unit. Study patients (*n =* 112) meeting the pre-set criteria were assessed by the principal investigator (PI) and a registered nurse (RN, *n =* 18). Internal consistency, inter-rater reliability, and concurrent validity of the scales were calculated and face validity and usability evaluated.

**Results:**

Internal consistency was from .76 to .86 for all three scales. Inter-rater reliability between PI and RNs was .87 with NEECHAM, .60 with CAM and .47 with Nu-DESC. Concurrent validity was .56 and .59 between CAM and NEECHAM, and .68 and .72 between NEECHAM and Nu-DESC. In the PI group, the correlation between CAM and Nu-DESC was .91, in the RN’s group .42. Nu-DESC was evaluated as the most usable scale.

**Conclusion:**

The findings strengthen the earlier research on the scales and indicate that the Finnish NEECHAM and Nu-DESC correlates with CAM algorithm and with each other. They seem to be clinically viable in assessing patients’ delirium in surgical wards but more validity testing is needed.

## Background

Delirium is a severe clinical problem for patients and their relatives as well as for health care professionals. It causes human suffering, a lower quality of life, lengthens hospital stays, leads to institutionalization, and increases mortality and costs [1, 2]. Delirium can occur in patients of all ages but is most common in patients over 65 years of age. According to literature, the incidence of delirium varies from 20% and even up to 79% in hospitalized older patients [3].

Based on the Diagnostic and Statistical Manual of Mental Disorders (DSM-IV, The American Psychiatric Association, APA) delirium diagnostic criteria are: 1) disturbance of consciousness, 2) a change in cognition or the development of a perceptual disturbance, 3) the disturbance develops over a short period of time and fluctuates, and 4) there is evidence that the disturbance is caused by the direct physiological consequences of a general medical condition [4]. In The International Classification of Diseases (ICD-10) the definition has expanded with disturbances in psychomotor behaviour, emotion and the sleep-wake cycle [5].

Due to the fluctuation of symptoms, nurses are in a key position in observing the patient, identifying and communicating the relevant symptoms to the physician, and managing the patient’s state. All symptoms of delirium are important for recognition, because they can lead to earlier detection and management of the state. Unfortunately, nurses in some settings may lack understanding and knowledge of the symptoms and state [6] and may not be able to recognize it in regular care [7]. Consequently, the documentation of delirium in the patients’ charts is insufficient [8] and, in nurses’ notes, the most common comment entered is “confusion”, without any specific symptom notes [9, 10]. For the recognition of the state of delirium, the routine use of formal instruments is recommended [11]. The assessment scale must be feasible and accepted by nursing staff [12].

In this study, the interest was to find a usable scale for assessing symptoms of delirium among Finnish patients following surgery and to help nurses detect the early signs of the state. The aim was to compare the consistency of the measurements with different instruments. The study process started with a literature search to learn which scales to assess delirium already exist. A literature search was made without year or language limitations in August 2010 and August 2011 from the several databases.

Based on the literature, there were some scales with promising test results for the purpose of detecting the state, but only a few are ready to be used in clinical care [13]. The most widely tested, translated and applied assessment scale is the Confusion Assessment Method (CAM), which has been developed as based on literature review and expert consensus and validated against the DSM-III-R criteria of delirium by Inouye et al. (1990) [14] . After seven high-quality validation studies with over 1000 subjects, CAM effectively separated delirious and non-delirious patients with sensitivity (the proportion of patients with delirium who test positive) of 94% [95% CI: 91–97%] and specificity (the proportion of patients without delirium who test negative) of 89% [95% CI: 85–94%] [15]. The CAM algorithm is the only delirium assessment scale that has been translated into Finnish [16] and statistically tested amongst the Finnish population [17]. It showed sensitivity of 84% and specificity of 81% with DSM-IV criteria. This indicates that Finnish CAM is an acceptable screening instrument, but the diagnosis should be ensured with DSM-IV criteria of delirium [17]. Yet, there is international evidence that the CAM has low sensitivity in the use of the clinical nurses [18]. That is why in this study the interest was to find another reliable and feasible scale for clinical nursing practice. There is no golden standard for delirium assessment for clinical nursing. Hence, the most valid and reliable available instrument was selected to be the comparison tool and its use with the other chosen instruments was compared.

After the literature search, it was decided that three scales would be taken into further evaluation. These scales were the Neecham Confusion Scale (NEECHAM) [19], Nursing Delirium Screening Scale (Nu-DESC) [20], and Delirium Observation Scale (DOS) [21]. They all fulfilled the pre-set prerequisites which were based on Steiner and Norman’s criteria (when available) (2008) [22] and are presented in Table 1. The Criteria were: 1) the scale has been developed for adult patients with delirium, 2) the scale has been developed for the use of nurses, 3) the scale has been tested in an acute nursing environment, 4) scientific articles concerning the development and testing of the scale were available, 5) the psychometric properties of the scale were available, 6) the scale has been validated in at least one foreign language to indicate cultural sensitivity, and scientific articles describing the validation process were available. After comparing the scales, NEECHAM and Nu-DESC were chosen for the validation process.

**Table 1:** Criteria and scoring for considered scales

Criteria and scoring	NEECHAM	Nu-DESC	DOS
Context: 1 = Acute care environment 0 = other	1	1	1
Assessor: 1 = non-expert 0 = expert (e.g. physician)	1	1	1
Amount of patients under assessment 2 = over 150 1 = 100–150 0 = under 100	2	2	2
Usability 2 = short, incl. filling and scoring instructions 1 = long, incl. filling and scoring instructions 0 = long or no instructions	1	2	1
Process of development based on: 2 = DSM-III/ IV and is reported 1 = other and is reported 0 = no report of development	1 Delphi panel, correlates with DSM-III-R	2	2
Correlation validity: correlation to the DSM-criteria 2 = r >0.60 1 = 0.40 < r < 0.60 0 = r < 0.40 or not reported	1 0.70, 0.54^3^	2 0.71^4^	0 not reported
Concurrent validity: Correlation to the comparison scale 2 = r > 0.60 1 = 0.40 < r < 0.60 0 = r < 0.40 or not reported	2 0.87 MMSE^1,3^	2 0.67 MDAS^2,4^	2 0.63 CAM^5^ 0.79 MMSE^1,5^
Discriminant validity: 1 = appropriately discriminates confusion from non-confusion 0 = no discrimination or not reported	1 sensitivity 1.0^6^ specificity .87^6^	1 sensitivity .86^4^ specificity .87^4^	1 sensitivity .89^6^ specificity .88^6^
Internal Consistency: Cronbach’s α 2 = 0.70 < α < 0.90 1 = α > 0.9 or 0.60 < α < 0.70 0 = α < 0.60 or not reported	2 0.90^3^	0 not reported	1 0.93^5^
Interrater reliability: Cohen’s κ 2 = > 0.8 1 = 0.6 < κ < 0.8 0 = < 0.6 or not reported	1 0.65^3^	2 0.89^4^	0 not reported
Amount of validation to other languages 2 = 2 or over 1 = 1 0 = none or not mentioned	2	2	0
Total	13	17	11

^1^Mini-Mental State Exam, ^2^The Memorial Delirium Assessment Scale, ^3^[19], ^4^[20], ^5^[27], ^6^[39]

### Aim and objectives

In this study, the interest was to find a scale to be used in assessing symptoms of delirium among Finnish patients following surgery and to help nurses detect the early signs of the state. The aim of this study was to psychometrically test the Finnish versions of the Neecham Confusion Scale (NEECHAM) and the Nursing Delirium Screening Scale (Nu-DESC) in surgical nursing care using the Confusion Assessment Scale (CAM) algorithm as a comparison scale. The detailed objectives were to explore 1) the internal consistency and inter-rater reliability, 2) the concurrent and face validity, and 3) the usability of the Finnish versions of the scales.

## Methods

### Study protocol and design

**Fig. 1:**
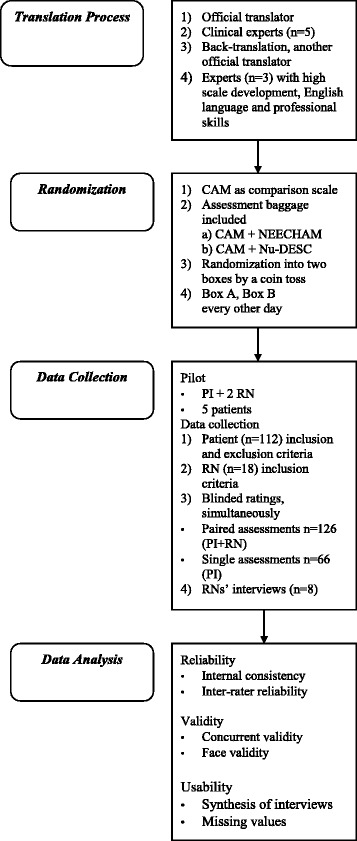
Study Protocol

The reliability, validity and usability of the Finnish versions of the NEECHAM and Nu-DESC scales in surgical nursing care were evaluated. The study included translation, piloting, clinical testing, statistical analysis and usability assessment of the scales. The study protocol is shown in Fig. 1. This randomized, blinded, instrument testing study was conducted at one university hospital in one vascular surgery unit. Data were collected between April and November 2011 (first data collection), and completed in November 2012 (additional data collection).

## The study

### Instruments

Three separate scales were used in the study. The CAM algorithm was used as a comparison scale. It has four items and categorizes patients into those who may have and those who may not have delirium. It poses questions regarding: 1) acute onset and fluctuating course, 2) inattention, 3) disorganized thinking and 4) altered level of consciousness. The first question requires information about the patient’s previous mental state, which can be retrieved, for example, from the patient’s family or patients’ medical charts. According to the CAM: Training Manual and Coding Guide, delirium is suggested if items 1 and 2 are positive at the same time with either 3 or 4 [23].

The NEECHAM has been developed by Neelon, Champagne, Carlson, and Funk (1996) based on DSM-III criteria and Delphi panel. The initial two-part study was conducted in an acute medical ward with elderly patients (the first part with 168 patients and the second part with 258 patients). Internal consistency was high in both parts (Cronbach’s alpha, α = .90). Inter-rater reliability was evaluated in the second part with a Pearson correlation coefficient (r = .91), and with Cohen’s kappa (κ = .65). Concurrent validity with a Pearson r was -.70 in the first part and -.54 in the second part with DSM-III-R diagnosis of delirium and .87 with the Mini-Mental State Exam (MMSE, first part) [19]. For example, NEECHAM has been validated in the Netherlands [24] as well as in Sweden [25]. It is designed to be used in daily nursing care of elderly patients hospitalized for acute medical illness [19].

The NEECHAM scale contains three subscales: processing, behaviour and physiological status. Subscale “processing” focuses cognition like attention, recognition and action on command, in addition to memory and orientation. Subscale “behaviour” focuses on physical performance such as appearance control, sensor motor performance, and verbal manifestation. Subscale “physiological status” includes vital functions, oxygenation, and continence. It requires measurements of blood pressure, heart rate, oxygen saturation, breathing rate and body temperature. All subscales are divided into three questions. In total, the scale includes nine items. In one item, there are from three to six options which are rated from 0 to 5. Overall, the scores may range from 0 to 30. Scores 0 to 19 indicate acute and moderate confusion, 20–24 indicate mild confusion, 25 to 26 indicate risk for confusion, and scores equal or over 27 indicate normal status. The cut-off score is 24 [19].

The Nu-DESC was developed for nurses by Gaudreau, Gagnon, Harel, Trembly, and Roy (2005) and is based on the Confusion Rating Scale (CRS) developed by Williams, Ward and Campbell (1988) [20]. The original CRS did not sufficiently separate all dimensions of delirium which raised a need for scale improvement [26]. The Nu-DESC was initially tested at the hemato-oncology and internal medicine units with 146 patients. Inter-rater reliability with Cohen’s kappa was .89 (95% CI, 0.75–1.0) with CAM. Concurrent validity with Pearson r was .71 with DSM-IV criteria and .67 with the Memorial Delirium Assessment Scale (MDAS). The sensitivity was 85.7% and specificity 86.8% [20]. The Nu-DESC has been validated in Germany [27] and in China (Hong Kong) [28]. The Nu-DESC is an observational five-item scale with the following items: disorientation, inappropriate behaviour, inappropriate communication, illusions/hallucinations, and psychomotor retardation. Each item is rated from 0 to 2 according to the presence and intensity of each symptom. This brings maximal scores up to 10. Scores over 2 indicate that there is 86% probability that delirium is present [20].

The translation process of the NEECHAM and Nu-DESC scales was based on a review by Maneesrivongul and Dixon (2004) [29]. The process included translation and back-translation, and both official translators and bilingual experts were used (Fig. 1). Cultural and semantic equivalences of the source and target versions were examined. The translation process consisted of: 1) translation from English into Finnish by one official translator, 2) evaluation of the semantic and clinical relevance of the translations by experienced nurses (*N =* 5), 3) back-translation from Finnish into English by another official translator, and 4) comparison of the back-translated scales with the original ones by three experts with expertise in scientific, scale development, and English language. During the process, minor changes in the Finnish phrasing were made to ensure the clinical understandability of the scales.

### Sample

The sampling included two groups: 1) adult patients following surgery who were the objects of the assessments and 2) registered nurses (paired with the principal investigator (PI)) who tested the scales and assessed the patients. Patients with fluent Finnish and who had undergone a vascular arterial surgical procedure were included. The exclusion criteria were: carotid arterial procedure; a diagnosed memory, mental health or neurological disease; difficult seeing or hearing disability; alcohol or drug abuse; and postoperative intensive care. The exclusion criteria were set because the testing of the assessment scales required patient participation and verbal interaction. It was found important to not mix up chronic diseases like dementia with the phenomenon under study. Patients with re-operations were included. The basic patient characteristics (age, gender, weight, length, American Society of Anesthesiologists Physical Status classification (ASA), type of hospital admission and medical diagnosis as well as surgical and anaesthesia procedures) were collected from the patients’ medical charts by the PI.

Sample size calculation was based on McNemar’s test, which can be used in dichotomous parameters. Sample size of 105 paired assessments has a power of 80% to detect a 15% difference in discordant pairs with a test significant level of 0.05. This was considered the minimum rather than the maximum size of various assessment groups.

A total of 18 Finnish-speaking registered nurses (RNs) with at least three years of clinical experience participated in the study. They were educated to use the assessment scales by the PI. The training consisted of oral and written information about delirium and instructions in how to use the scales that were to be tested. The training of the PI was based on the Training Manual and Coding Guide of the CAM [23].

### Procedure

The scales were piloted in one surgical ward with two RNs and five patients not participating in the study. The pilot included 15 paired observations by both the PI and a RN. Based on the results from the pilot study, minor changes were made to the outline of the assessment scales, for example, enlarging the font size, and some clarifications to the instructions on how to fill in the forms.

There were two kinds of assessment form packages (CAM & NEECHAM and CAM & Nu-DESC). The PI used simple randomization by tossing a coin to raffle the assessment form packages into two boxes [30]. First the box A was filled and then box B. Every even day RNs took assessment forms from box A and every odd day from box B. The PI acted the other way around. In that way, the patient was always assessed with four scales, two assessments with CAM (one by the PI and one by the RN), and two assessments with NEECHAM or Nu-DESC, randomly applied. As a result of the randomization, it was possible that both the PI and the RN took NEECHAM or Nu-DESC, or they took a different scale. The fluctuation of the phenomenon allowed for the same patient being assessed on different days. Thus, the patients were assessed once a day on the first, second, and/or third postoperative day between 8 am and 2 pm always by the PI and one RN. Ratings between the assessors were blinded.

The assessors simultaneously observed and interviewed the patients in authentic nursing situations, e.g. during wound treatment. Standardized questions were not used. Instead, the patients were encouraged to explain their current whereabouts, the date, events prior to the hospitalization and during the current day and circumstances at home, etc. The required physiological measurements (BP, HR, temperature, oxygen saturation, and breathing frequency) were performed with a NoNin Medical Onyx saturation meter, Omron M-6 sphygmomanometer and Braun type 6021 ear thermometer. Also, nurses’ notes in the medical charts about the patients’ previous condition were examined. The first part of the data collection was stopped after 110 paired assessments. The additional data collection period consisted of 16 paired assessments and was needed in spite of randomization, due to the lack of assessments with the Nu-DESC scale.

After the first data collection, those RNs (*n =* 8) who were willing to participate were interviewed about the usability of the scales. The semi-structural interview consisted of questions about the clarity and understandability of the scales, the variables and items in them as well as the potential scoring difficulties. Also, nurses were asked which scale they considered to be the most useful. Furthermore, nurses’ general opinions about the systematic assessment of delirium and their familiarity with other clinical rating scales were investigated. In addition to RNs’ free expressions, yes and no answers were required to get quantified data of their opinions. The interviewed RNs (*n =* 8) had work experience as nurses totalling between eight and fifteen years each. They were familiar with clinical assessments scales, such as pain assessment scales and patient classification scales.

### Statistical measures

Reliability of all used scales was calculated and tested with internal consistency (how well the items correlate with each other; [31] and inter-rater reliability (how similar the results between two or more independent assessors are when they assess the same target at same time; [32]. Internal consistency was determined by calculating Cronbach’s alpha (α) with recommendable values between .7 and .9 [33]. Inter-rater reliability was calculated with Cohen’s kappa (κ), comparing the PI’s ratings to the RNs’ ratings with every scale separately. Interpretation of the values followed the guidelines by Landis and Koch (1977), according to which agreement between observers is almost perfect if kappa is between .81 and 1.0, substantial between .61 and .80 and moderate between .41 and .60 [34]. Kappa values are reported as estimates together with confidence intervals [35] and *p*-values.

Validity denotes the ability of the instrument to measure the attributes of the phenomenon under study, and this was tested with concurrent validity (how well the scores in the scale under testing correlate with scores in the comparison scale) and face validity (how well the instrument seems to measure the phenomenon under study) [36]. Concurrent validity was calculated using CAM algorithm as the comparison scale and also by comparing the NEECHAM and Nu-DESC scales with each other with the Spearman’s Rank Correlation Coefficient (r_s_).

Correlation was separately calculated between CAM and NEECHAM and CAM and Nu-DESC from the PI and RN assessments. Also, correlation between NEECHAM and Nu-DESC was calculated from the paired assessments where PI had either NEECHAM or Nu-DESC and RN had the opposite form. Face validity was assessed by the experts taking part in the translation process, calculating the missing values of filled forms, and by interviews of RNs participating in the study. Usability was assessed as based on the RNs’ individual, semi-structured interviews and data were analysed by calculating the amount of yes and no answers and making a synthesis of the free responses to interview questions. Statistical data were analysed using the SPSS version 19.

## Results

In all, PI made 192 and RNs made 126 assessments. Thus, there were 318 individual assessments by PI or a RN and, in total, 126 paired assessments by a RN and the PI. In addition, the PI made 66 assessments alone (i.e., the nurse involved in the nursing situation was not enrolled in the study). These assessments were included in the data as single assessments by the PI. The numbers of filled, accepted and excluded forms are shown in Table 2. Excluded forms included lost, blank and partly filled forms. Altogether, three assessment form packages filled by RNs were lost and six CAM and NEECHAM packages were returned blank. There is no information available of the missed patients or of the lost packages. However, 117 paired assessments were included in the statistical analysis. Furthermore, a total of seven NEECHAM and two Nu-DESC assessment forms were only partly filled and therefore excluded, but assessments with CAM were included in the analysis.

**Table 2:** Number of filled, accepted, and excluded assessments by PI and RN

	CAM			NEECHAM			Nu-DESC		
	Total	Accepted	Excluded	Total	Accepted	Excluded	Total	Accepted	Excluded
PI	192	191	1	80	78	2	112	111	1
RN	126	117	9	54	44	10	69	68	1
Total	318	308	10 (3%)	134	122	12 (9%)	181	179	2 (1%)

*PI* Primary Investigator, *RN* Registered Nurse

In total, 112 patients were assessed. Their demographics are presented in Table 3. A typical patient was a slightly overweight male aged 77 with three co-morbid diseases, and classified to an ASA 3 category. The incidence of positive findings in PI’s CAM assessments (*n =* 191) was 14.6%.

**Table 3:** Demographics of the study patients (*n =* 112)

Demographics of the study patients	*n*	%	Cumulative %
Gender			
Male	61	54.5	54.5
Female	51	45.5	100.0
Type of admission			
Elective	64	57.1	
Emergency	47	42.0	99.1
Transfer from another hospital	1	0.9	100.0
ASA classification			
1-2 (normal healthy patient or a patient with mild systemic disease)	1	0.9	
3 (a patient with severe systemic disease or a patient over 65 years)	56	50.0	50.9
4-5 (a patient with severe systemic disease that is a constant threat to life or a moribund patient who is not expected to survive without the operation)	30	26.8	77.7
Missing	25	22.3	100.0
Number of co-morbidies			
0-1	15	13.4	
2-3	42	37.5	50.9
4-5	42	37.5	88.4
6-7	13	11.6	100.0
BMI			
Under normal (<18,5)	4	3.6	
Normal weight (18.5-25)	44	39.3	42.9
Slightly overweight (25.1-30)	37	33.0	75.9
Overweight (30.1-35)	17	15.2	91.1
Difficult overweight (35.1-40)	3	2.7	93.8
Morbid obesity (>40)	1	0.9	94.6
Missing	6	5.4	100.0
Wound classification			
Clean	88	78.6	
Clean contaminated	5	4.5	83.1
Contaminated	13	11.6	94.7
Dirty	4	3.6	98.3
Missing	2	1.7	100.0
Top three main diagnosis			
Atherosclerosis of arteries of extremities (I70.2)	67	59.8	
Abdominal aortic aneurysm without rupture (I71.4)	17	15.2	75.0
Embolism and thrombosis of arteries of the lower extremities (I74.3)	6	5.4	80.4
Other	22	19.6	100.0
Top three main procedures			
Angiography to lower limb arteries	23	20.5	
Stent-graft replacement to abdominal aortic aneurysm (PDQ05)	16	14.3	34.8
Femoro-popliteal by-pass (PEH56/ PEH57)	14	12.5	47.3
Other	59	52.7	100.0

*ASA* American Society of Anesthesiologists Physical Status classification, *BMI* Body Mass Index

The results are not presented per patient but per assessment. Half of the patients were assessed two times -i.e., in two different days-, 39% one time and 11% three times. 57% of assessments were made on the patients’ first post-operative day, 24% on the second and 19% on the third post-operative day. Cross-tabulations of positive (delirium exists) and negative (normal) findings of paired assessments by scale and by rater (PI vs. RN) are presented in Table 4. The lowest amount of findings where both the PI and RN had a positive result was found with CAM (7.7%) and Nu-DESC (8.1%).

**Table 4:** Crosstabulation of positive and negative findings in paired assessments by PI and RNs

CAM		RNs		
		Negative *n* (%)	Positive *n* (%)	Total *n* (%)
PI	Negative	98 (83.8)	2 (1.7)	100 (85.5)
	Positive	8 (6.8)	9 (7.7)	17 (14.5)
	Total	106 (90.6)	11 (9.4)	117 (100.0)
NEECHAM				
PI	Negative	13 (68.4)	0 (0.0)	13 (68.4)
	Positive	1 (5.3)	5 (26.3)	6 (31.6)
	Total	14 (73.7)	5 (26.3)	19 (100.0)
Nu-DESC				
PI	Negative	29 (78.4)	3 (8.1)	32 (86.5)
	Positive	2 (5.4)	3 (8.1)	5 (13.5)
	Total	31 (83.8)	6 (16.2)	37 (100.0)

Internal consistency of the scales, inter-rater reliability, and concurrent validity between the scales and assessor groups are presented in Table 5. Instead of the minimum sample size, we took the maximum amount of filled forms – i.e. all single and paired assessments – into the statistical analyses. The lowest alpha value (.76) was found in the Nu-DESC in the PI assessments. The highest Kappa value was with the NEECHAM (.87) and lowest with the Nu-DESC (.47). The Spearman’s Rank Correlation Coefficients were most divided between PI and RNs with the Nu-DESC (.91, .42).

**Table 5:** Psychometric properties of tested scales

	Internal consistency^1^	Inter-rater reliability^2^	Concurrent validity^3^
CAM	PI (*n =* 191) 0.83 (0.792, 0.867)	PI vs. RNs (*n =* 117) 0.60 (0.374, 0.820) (*p =* 0.000)	
	RNs (*n =* 111) 0.86 (0.818, 0.899)		
NEECHAM	PI (*n =* 75) 0.80 (0.725, 0.862)	PI vs. RNs (*n =* 19) 0.87 (0.631, 1.113) (*p =* 0.001)	
	RNs (*n =* 42) 0.80 (0.697, 0.880)		
Nu-DESC	PI (*n =* 111) 0.76 (0.680, 0.823)	PI vs. RNs (*n =* 37) 0.47 (0.071, 0.863) (*p =* 0.022)	
	RNs (*n =* 69) 0.78 (0.688, 0.853)		
NEECHAM (PI) vs. Nu-DESC (RNs)			PI vs. RNs (*n =* 28) 0.68 (*p <* 0.01)
Nu-DESC (PI) vs. NEECHAM (RNs)			PI vs. RNs (*n =* 25) 0.72 (*p <* 0.01)
CAM vs. NEECHAM			PI (*n =* 80) 0.56 (*p <* 0.01)
			RNs (*n =* 44) 0.59 (*p <* 0.01)
CAM vs.Nu-DESC			PI (*n =* 112) 0.91 (*p <* 0.01)
			RNs (*n =* 66) 0.42 (*p =* 0.01)

^1^Cronbach’s α, (95% CI)

^2^Cohen’s κ, (95% CI, *p*-values)

^3^Spearman’s Rank Correlation Coefficient r_s_ (*p-*values)

Face validity of the scales was partly evaluated by the missing values. In NEECHAM, there were missing values both in the PI and RN assessments, mostly with physiological parameters which did not affect the rating of patients with or without delirium. The interviewed RNs believed that, on the whole, the scales were able to measure the patients’ symptoms of delirium. All scales were easy and quick to fill in (less than 5 min), and the scale variables were clear and understandable. Nu-DESC was evaluated as the most usable scale. However, it obtained critique with regard to the sliding scoring categories (1 = mild to moderate, 2 = moderate to severe). NEECHAM was criticized for being too long (two pages) and time-consuming, even if it was easy to understand and fill in because the alternatives of the questions were easy to select. In the use of CAM there were some difficulties to separate symptoms of inattention and of disorganized thinking.

## Discussion

This study confirmed the applicability of the Neecham Confusion Scale and the Nursing Delirium Screening Scale in the Finnish surgical patient care context. The validation process of the Finnish versions of the scales was completed in the study. Both reliability and validity were evaluated. Current Finnish versions of the NEECHAM and the Nu-DESC scales seem to be reliable and practical to perform within the nursing environment where the study was conducted. Validity of the tested scales seems to need more evidence. Furthermore, as a secondary finding of the study, more information concerning the reliability of the Finnish version of the CAM algorithm within the same nursing context was attained.

Internal consistency was within the recommended values of Cronbach’s alpha (.70–.90) for all three scales in both PI and RN assessment groups [33]. NEECHAM alpha values in the PI and RN group were a little lower than in the initial study (α = .90) [19] and in the validation studies of Flemish (α = .88) [24] and Swedish (α = .83) [37] scales. The Nu-DESC alpha values were more appropriate than in the studies on the German [27] and Chinese [28] versions, where alpha values were over the limit .90. In this study, CAM alpha values were good (PI α = .83, RN α = .86). There are no previous statistics available about the internal consistency of the Finnish version of CAM.

Inter-rater reliability between PI and RNs was almost perfect (.87) with NEECHAM, but moderate (.47) with Nu-DESC and (.60) with CAM. The NEECHAM kappa value was better than in the Flemish version (κ = .65, [24]. The Nu-DESC kappa value was comparable with the Chinese version (κ = .52, [28], but lower than in the initial study (κ = .89, [20] and in the German version (κ = .83, [27]. As seen in the Table 5, the confidence interval of kappa with NEECHAM was from .631 to 1.113 but with CAM and Nu-DESC the range was much lower and wider. In the NEECHAM data there were small amount of paired assessments. In the Nu-DESC and in the CAM data, there were a low number of patients with delirium. These may exert an impact on the kappa values of all scales. Inter-rater reliability for all three scales was significant (*p <* .05). However, when interpreting the results it needs to be noticed that a potential statistical significance means only little if the kappa value is under .60. [38].

Concurrent validity between the CAM and the NEECHAM showed positive correlation (r_s_ = .56, .59) in both assessor groups. Surprisingly, it varied between CAM and Nu-DESC by the assessor: in the PI assessment group, the correlation was strong (r_s_ = .91, *p <* .01), but in the RN group only moderate (r_s_ = .42, *p =* .01). This may be explained by the RN feedback, stating that the Nu-DESC was easy to fill in, but the categories were not clear enough. Also, there may have been too little attention given to the training for use of Nu-DESC. Correlation between NEECHAM and Nu-DESC was .68 in the PI assessment group and .72 in the RN group.

All the Spearman’s Rank Correlation Coefficient values with all scale pairs were significant (*p* ≤ .01).

The Nu-DESC scale was the most popular amongst the RNs interviewed. All scales were evaluated as usable in assessing patients with delirium. All scales were quick to fill in (from two to five minutes). However, the NEECHAM scale received critique regarding the length and documentation of the physiological parameters, which easily lead to missing values or, on the other hand, repetitive documentation, both in the scale and the medical chart. It can be suggested that this study broadens the menu of potential assessment scales for delirium.

Multiprofessional approach is needed in the care of delirious patients. Both nurses and physicians are needed to detect and manage patients’ state because of the acute and fluctuating onset. Nurses’ task is to detect and evaluate early signs of patients’ symptoms. Usually the interventions to help patients’ state are made as based on both nurses’ assessments and physicians’ diagnoses. Reliable and valid instruments will help the assessment and diagnosing of the patients’ state which will improve patients’ outcomes.

### Limitations

The data are not strongly in support of the concurrent validity of any of the scales and only with the NEECHAM the evidence of reliability is convincing. In the RN group, there were a rather small number of assessments with NEECHAM and Nu-DESC, obviously affecting the results. Inter-rater reliability with the kappa can be calculated for rather small sample sizes but when calculating the confidence intervals, there should not be less than 30 comparisons [31]. It must be stated that only CAM received the total of 105 paired assessments calculated as being sufficient by McNemar’s test before data collection.

The approach to use the CAM algorithm as a comparison scale instead of expert diagnosis was based on the focus of the study that was to assess scales in the use of clinical nurses because nurses do not diagnose the phenomena but rather evaluate the adequate symptoms. Although there is no golden standard measurement in Finnish delirium assessment, the CAM is widely tested and used over the world and the CAM algorithm is translated to Finnish and tested in Finnish hospital care.

The study was conducted in two parts due to the lack of patients with delirium in the Nu-DESC group during the first part of data collection. The patient exclusion criteria were strict. Patients with dementia or cognitive impairment were excluded to make the scale testing more reliable. It may have also been better if patients who had been in the intensive care unit had been included in the study. These criteria may have had an effect on the total number of patients with delirium and, thus, on the results.

In spite of the aforementioned deficiencies in the reliability and validity results, Nu-DESC and NEECHAM were usable and feasible among clinical nurses. Therefore, this study broadens the menu of potential assessment scales for delirium in surgical units. The staff needs to be able and willing to fit an assessment scale into their busy work flow. Reliability and validity of a scale should be considered with psychometric parameters and also in the light of the clinical context.

In this study, RNs were used as assessors in order to mimic real-life clinical situations. It is suggested that this increased the validity of the research and made the research design more comfortable for the patients. The evaluation of the patients’ state in a clinical nursing situation is an adequate method to obtain enough information needed to fill in assessment scales for delirium. During the study, this point of view was based on the RNs’ interviews together with experiences in assessment situations. Also, statistical analyses of psychometric properties of the scales support this conclusion. Moreover, using the real-life situations in the study, the results are easy to adopt in practice. However, the use of RNs instead of a research assistant complicated data collection. There were situations where the RN enrolled in the study could not assess the patient, due to other working tasks or different working hours. The study was conducted so, that the care of other patients or the work of other employees was not disturbed.

## Conclusion

In summary, this study explored the reliability, validity and usability of NEECHAM and Nu-DESC in Finnish surgical nursing care, using the CAM algorithm as a comparison scale. The validation process indicates that the Finnish version of NEECHAM is reliable and usable in its current version in clinical practice to assess patients’ delirium at vascular surgical wards. The usability of the Finnish Nu-DESC was evaluated as being the best but its reliability needs more testing. The results concerning validity show that there is correlation between all scales. However, more validity testing is needed with all scales used in this study. The use of an assessment scale can help nurses to recognize patients’ delirium. Registered nurses are able to assess patients’ delirium based on clinical care situations, but training in how to use the scales must be taken into account.

## Abbreviations

ASA: American Society of Anesthesiologists Physical Status classification
CAM: Confusion Assessment Method
DOS: Delirium Observation Scale
DSM: the Diagnostic and statistical Manual of Mental Disorders
MMSE: Mini-Mental state exam
NEECHAM: Neecham Confusion Scale
Nu-DESC: Nursing Delirium Screening Scale
